# Supplementary material for: Economic Evaluation of Direct Oral Anticoagulants Compared to Warfarin for Venous Thromboembolism in Thailand: A Cost-Utility Analysis
Source: Int J Environ Res Public Health. 2023 Feb 11;20(4):3176. doi: 10.3390/ijerph20043176 (PMC9961808; doi:10.3390/ijerph20043176)
Supplement: Supplementary file 1 [file ijerph-20-03176-s001.zip › ijerph-2117410-supplementary.pdf]

SUPPLEMENTARY MATERIALS

Table S1: Clinical parameters, costs, and utilities

Parameter	Value	Range	Distribution	Source
Transition probabilities of patients receiving warfarin				
Recurrent PE	0.0300	0.0256-0.0346	Beta	[1]
Recurrent DVT	0.0140	0.0119-0.0162	Beta	[1]
Recurrent VTE after treatment discontinuation	0.0529	0.0450-0.0608	Beta	[2]
Probability of major bleeding				
ICH	0.0021	0.0017-0.0024	Beta	[3]
GI	0.0142	0.0120-0.0163	Beta	[3]
CRNM bleeding	0.0804	0.0683-0.0924	Beta	[1]
Probability of death due to major bleeding				
ICH	0.1439	0.1223-0.1654	Beta	[4]
GI	0.0978	0.0831-0.1124	Beta	[1]
CTEPH after acute PE	0.0161	0.0137-0.0186	Beta	[5]
CTEPH after recurrent PE	0.0289	0.0246-0.0333	Beta	[5]
Efficacy parameters				
Risk of ICH between DOACs and warfarin				
Apixaban	0.50	0.13-2.01	Lognormal	[6]
Dabigatran	0.28	0.07-1.13	Lognormal	[7,8]
Rivaroxaban	0.10	0.01-13.08	Lognormal	[6]
Edoxaban	0.28	0.10-0.75	Lognormal	[6]
Risk of GI bleeding between DOACs and warfarin				
Apixaban	1.00	0.02-51.63	Lognormal	[9]
Dabigatran	1.41	1.05-1.91	Lognormal	[9]
Rivaroxaban	0.25	0.00-13.08	Lognormal	[9]
Edoxaban	1.50	0.83-2.74	Lognormal	[9]
Risk of CRNM bleeding between DOACs and warfarin				
Apixaban	0.48	0.38-0.61	Lognormal	[10]
Dabigatran	0.62	0.44-0.87	Lognormal	[7]
Rivaroxaban	1.02	0.89-1.18	Lognormal	[11]
Edoxaban	0.80	0.68-0.93	Lognormal	[12]
Risk of overall VTE recurrence and VTE-related death between DOACs and warfarin in index PE				
Apixaban	0.90	0.50-1.61	Lognormal	[10]
Dabigatran	0.87	0.47-1.62	Lognormal	[7]
Rivaroxaban	1.13	0.76-1.69	Lognormal	[11]
Edoxaban	0.73	0.50-1.06	Lognormal	[12]
Risk of overall VTE recurrence and VTE-related death between DOACs and warfarin in index DVT				
Apixaban	0.83	0.54-1.26	Lognormal	[10]
Dabigatran	1.23	0.79-1.92	Lognormal	[7]
Rivaroxaban	0.70	0.46-1.07	Lognormal	[11]
Edoxaban	1.02	0.75-1.38	Lognormal	[12]
HR of excess mortality				
Index VTE	4.41	3.63-5.36	Gamma	[13]
ICH	2.60	2.20-5.60	Gamma	[14]
CTEPH	1.30	0.98-1.73	Gamma	[15]
Age-specific mortality rate				

Parameter	Value	Range	Distribution	Source
60-64 year	0.0060		Fixed	[16]
65-69 year	0.0085		Fixed	[16]
70 years and over	0.0251		Fixed	[16]
Other clinical parameters				
Proportion of index PE	53.35%		Fixed	[1]
Proportion of index DVT	46.65%		Fixed	[1]
Drug price (unit price; THB)				
Apixaban (2.5 and 5 mg)	45.75		Fixed	[17]
Rivaroxaban (15 mg and 20 mg)	87.38		Fixed	[17]
Dabigatran (150 mg)	48.01		Fixed	[17]
Edoxaban (60 mg)	1.85		Fixed	[17]
Warfarin (3 mg)	95.23		Fixed	[17]
Enoxaparin (60 mg/0.6 ml)	241.82		Fixed	[17]
Sildenafil (20 mg) (only for CTEPH)	7.93		Fixed	[17]
Direct medical treatment costs (THB)				
PE	41,256	35,067 - 47,444	Gamma	[18]
DVT	5,507	4,681 - 6,333	Gamma	[18]
GI bleeding	8,982	7,635 - 10,329	Gamma	[19]
ICH	39,205	33,324 - 45,086	Gamma	[19]
Post ICH	4,952	4,209 - 5,695	Gamma	[19]
CRNM	781	663 - 898	Gamma	[19]
CTEPH	33,075	28,113 - 38,036	Gamma	[20]
**Diagnostic costs**				
PE	5,530	4,701 – 6,360	Gamma	[20]
DVT	2,130	1,811 – 2,450	Gamma	[20]
CTEPH	6,200	5,270 – 7,130	Gamma	[20]
GI bleeding	2,000	1,700 – 2,300	Gamma	[20]
ICH	35,000	29,750 – 40,250	Gamma	[20]
Direct non-medical costs (THB)				
Travel cost	157	133 - 180	Gamma	[21]
Food cost	58	49 - 66	Gamma	[21]
Utilities				
Baseline utility	0.944		Fixed	[22]
Acute ICH	0.330	0.140 – 0.530	Beta	[23]
Post ICH with disability	0.620	0.320 – 0.920	Beta	[24]
Acute GI bleed	0.650	0.490 – 0.860	Beta	[23]
CTEPH	0.650	0.400 – 0.890	Beta	[25]
Utility decrements associated with clinical events and use of anticoagulants				
DVT	0.110	0.000 – 0.310	Beta	[23]
PE	0.320	0.000 – 0.590	Beta	[23]
CRNM bleeding	0.005	0.000 – 0.020	Beta	[23]
DOACs	0.002	0.000 – 0.006	Beta	[26]
VKA	0.013	0.000 – 0.047	Beta	[26]

Abbreviations: PE, pulmonary embolism; DVT, deep vein thrombosis; VTE, venous thromboembolism; ICH, intracranial haemorrhage; GI, gastrointestinal; CTEPH, chronic thromboembolic pulmonary hypertension; DOACs, direct oral anticoagulants; VKA, vitamin K antagonist; CRNM bleeding, clinically relevant non-major bleeding; HR, hazard ratio; THB, Thai baht.

Table S2 Budget impact inputs

Inputs	Value	References
Deep vein thrombosis incidence	0.030%	[27]
Pulmonary embolism incidence	0.009%	[27]
Proportion of current DOACs prescription	9.30%	[28]
% annual uptake increment	5%	Assumption
Total population aged 60 or over	12,071,837	National Statistical Office

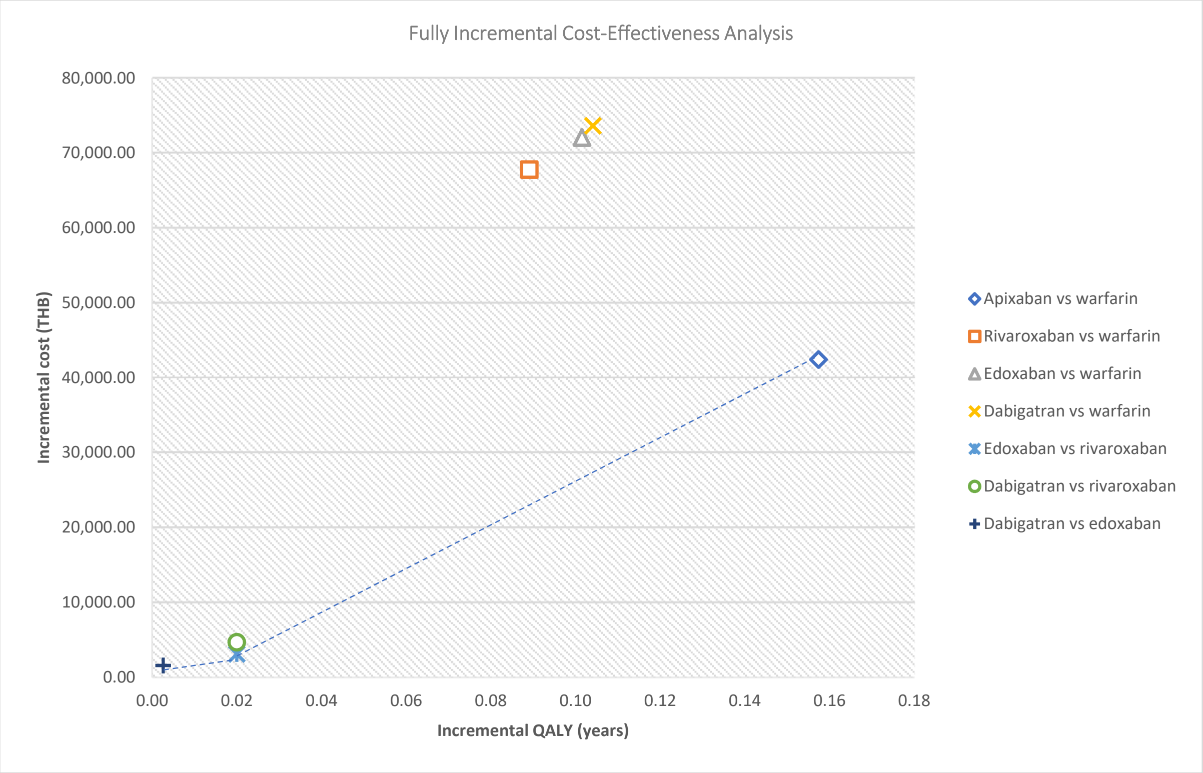

Figure S1: Fully incremental cost-effectiveness analysis
Abbreviations: THB, Thai baht; QALY, quality-adjusted life year.

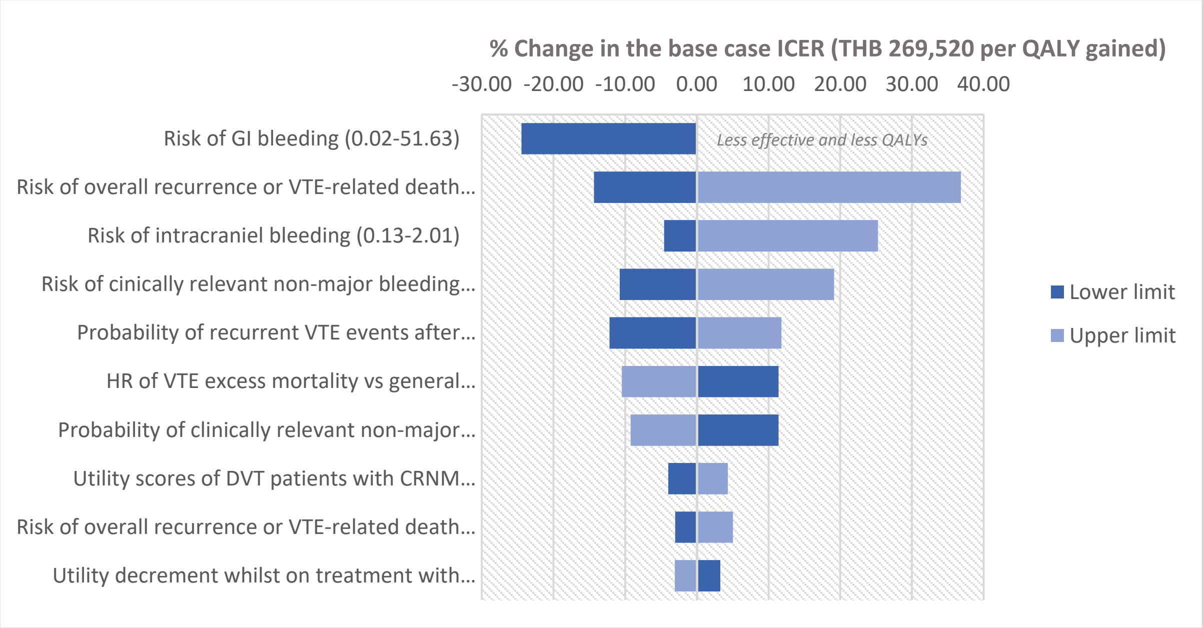

Figure S2 Tornado diagram for deterministic sensitivity analyses of apixaban versus warfarin
Abbreviations: ICER, incremental cost-effectiveness ratio; QALY, quality-adjusted life year; VTE, venous thromboembolism; PE, pulmonary embolism; DVT, deep vein thrombosis; VKA, vitamin K antagonist; HR, hazard ratio; GI, gastrointestinal; DOACs, direct oral anticoagulants.

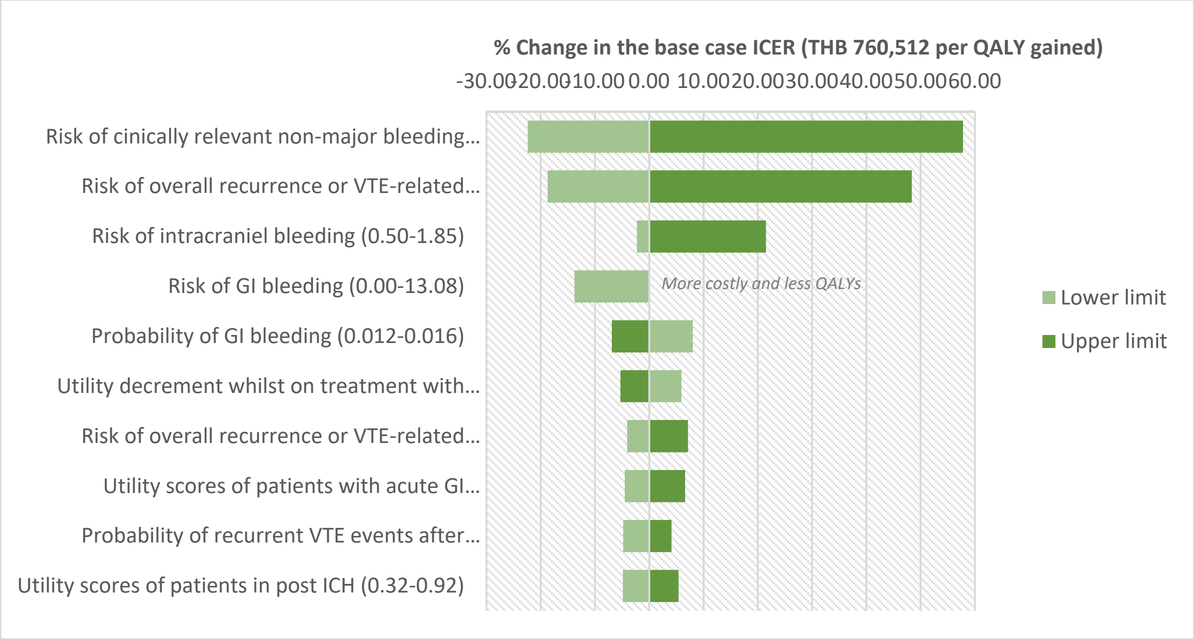

Figure S3 Tornado diagram for deterministic sensitivity analyses of rivaroxaban versus warfarin
Abbreviations: ICER, incremental cost-effectiveness ratio; QALY, quality-adjusted life year; VTE, venous thromboembolism; DVT, deep vein thrombosis; VKA, vitamin K antogonist; HR, hazard ratio; GI, gastrointestinal; ICH, intracraniel haemorrhage; CTEPH, chronic thromboembolic pulmonary hypertension.

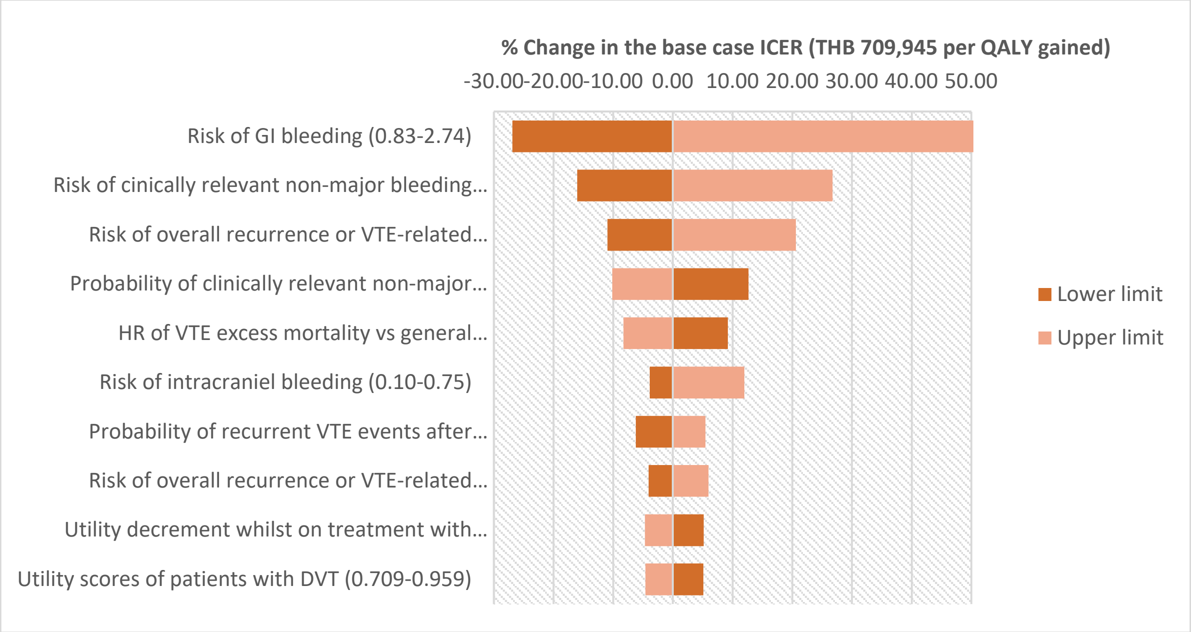

Figure S4 Tornado diagram for deterministic sensitivity analyses of edoxaban versus warfarin
Abbreviations: ICER, incremental cost-effectiveness ratio; QALY, quality-adjusted life year; VTE, venous thromboembolism; DVT, deep vein thrombosis; VKA, vitamin K antogonist; HR, hazard ratio; GI, gastrointestinal; PE, pulmonary embolism; CTEPH, chronic thromboembolic pulmonary hypertension; DOACs, direct oral anticoagulants.

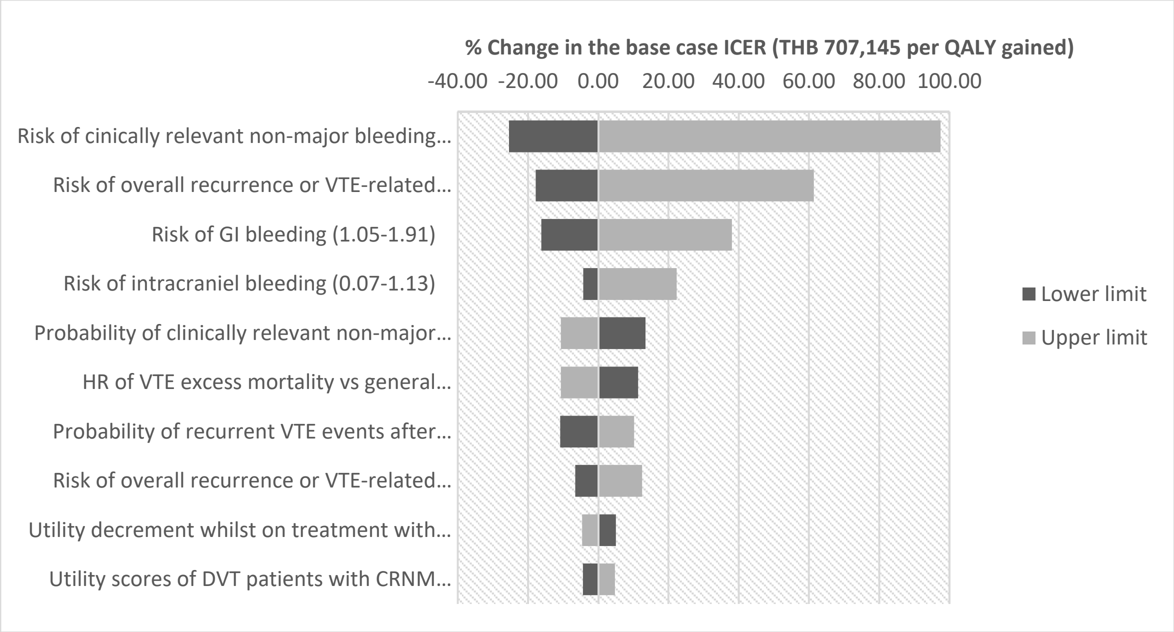

Figure S5 Tornado diagram for deterministic sensitivity analyses of dibagatran versus warfarin
Abbreviations: ICER, incremental cost-effectiveness ratio; QALY, quality-adjusted life year; VTE, venous thromboembolism; DVT, deep vein thrombosis; VKA, vitamin K antogonist; HR, hazard ratio; GI, gastrointestinal; PE, pulmonary embolism; DOACs, direct oral anticoagulants; ICH, intracraniel haemorrhage

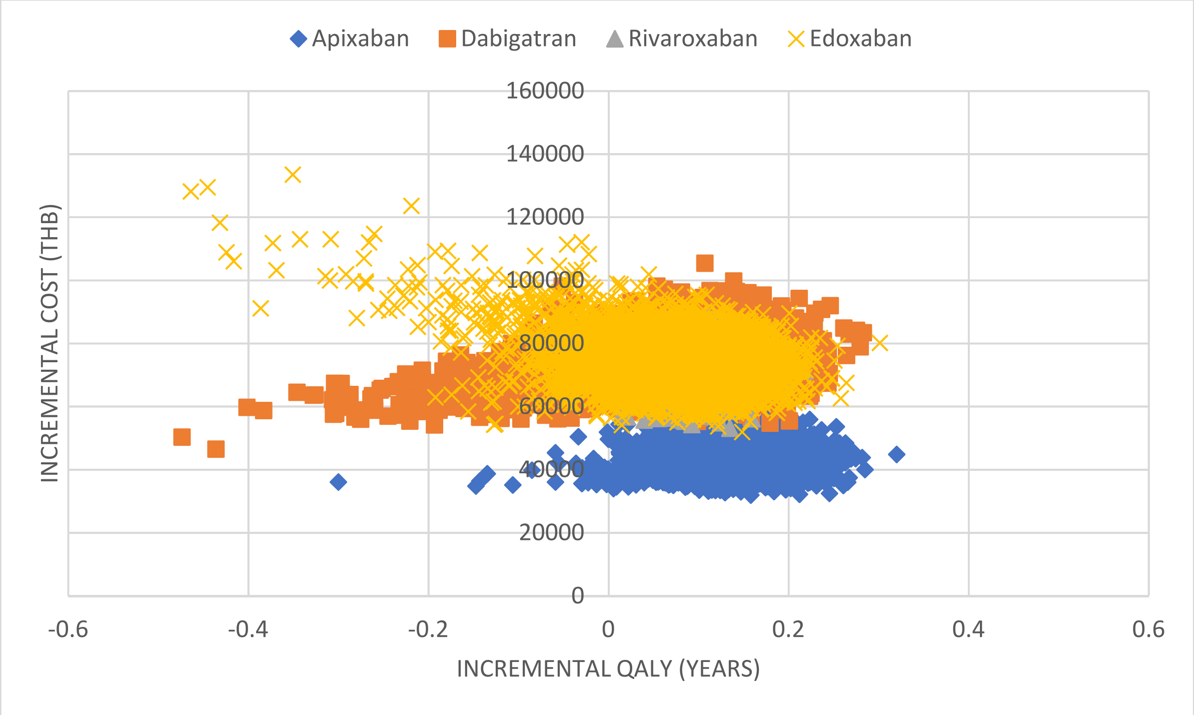

Figure S6: Probabilistic cost effectiveness plane results
Abbreviations: THB, Thai baht; QALY, quality-adjusted life year.

References

1. Nakamura M, Wang YQ, Wang C, et al. Efficacy and safety of edoxaban for treatment of venous thromboembolism: a subanalysis of East Asian patients in the Hokusai-VTE trial. *J Thromb Haemost*. Sep 2015;13(9):1606-14. doi:10.1111/jth.13055
2. Khan F, Rahman A, Carrier M, et al. Long term risk of symptomatic recurrent venous thromboembolism after discontinuation of anticoagulant treatment for first unprovoked venous thromboembolism event: systematic review and meta-analysis. *BMJ*. 2019;366:14363. doi:10.1136/bmj.l4363
3. Mitsuntisuk P, Nathisuwan S, Junpanichjaroen A, et al. Real-World Comparative Effectiveness and Safety of Non-Vitamin K Antagonist Oral Anticoagulants vs. Warfarin in a Developing Country. *Clin Pharmacol Ther*. May 2021;109(5):1282-1292. doi:10.1002/cpt.2090
4. Kongbunkiat K, Kasemsap N, Thepsuthammarat K, Tiamkao S, K S. National data on stroke outcomes in Thailand. *J Clin Neurosci*. Mar 2015;22(3):493-7. doi:10.1016/j.jocn.2014.08.031
5. Ende-Verhaar YM, Cannegieter SC, Vonk Noordegraaf A, et al. Incidence of chronic thromboembolic pulmonary hypertension after acute pulmonary embolism: a contemporary view of the published literature. *Eur Respir J*. Feb 23 2017;49(2):1601792. doi:10.1183/13993003.01792-2016
6. Wolfe Z, Khan SU, Nasir F, Raghu Subramanian C, B L. A systematic review and Bayesian network meta-analysis of risk of intracranial hemorrhage with direct oral anticoagulants. *J Thromb Haemost*. Jul 2018;16(7):1296-1306. doi:10.1111/jth.14131
7. Schulman S, Kakkar AK, Goldhaber SZ, et al. Treatment of acute venous thromboembolism with dabigatran or warfarin and pooled analysis. *Circulation*. Feb 18 2014;129(7):764-72. doi:10.1161/CIRCULATIONAHA.113.004450
8. Schulman S, Kearon C, Kakkar AK, et al. Dabigatran versus Warfarin in the Treatment of Acute Venous Thromboembolism. *N Engl J Med*. 2009;361:2342-2352.
9. Oh HJ, Ryu KH, Park BJ, BH Y. The risk of gastrointestinal hemorrhage with non-vitamin K antagonist oral anticoagulants: A network meta-analysis. *Medicine (Baltimore)*. 2021;100(11):e25216. doi:10.1097/MD.0000000000025216
10. Agnelli G, Buller HR, Cohen A, et al. Oral apixaban for the treatment of acute venous thromboembolism. *N Engl J Med*. Aug 29 2013;369(9):799-808. doi:10.1056/NEJMoa1302507
11. Prins MH, Lensing AW, Bauersachs R, et al. Oral rivaroxaban versus standard therapy for the treatment of symptomatic venous thromboembolism: a pooled analysis of the EINSTEIN-DVT and PE randomized studies. *Thromb J*. Sep 20 2013;11(1):21. doi:10.1186/1477-9560-11-21
12. The Hokusai-VTE Investigators. Edoxaban versus Warfarin for the Treatment of Symptomatic Venous Thromboembolism. *N Engl J Med*. 2013;369:1406-1415. doi:10.1056/NEJMoa1306638
13. Flinterman LE, van Hylckama Vlieg A, Cannegieter SC, FR R. Long-term survival in a large cohort of patients with venous thrombosis: incidence and predictors. *PLoS Med*. Jan 2012;9(1):e1001155. doi:10.1371/journal.pmed.1001155
14. Prandoni P, Trujillo-Santos J, Sanchez-Cantalejo E, et al. Major bleeding as a predictor of mortality in patients with venous thromboembolism: findings from the RIETE Registry. *J Thromb Haemost*. Nov 2010;8(11):2575-7. doi:10.1111/j.1538-7836.2010.04039.x
15. Ng AC, Chung T, Yong AS, et al. Long-term cardiovascular and noncardiovascular mortality of 1023 patients with confirmed acute pulmonary embolism. *Circ Cardiovasc Qual Outcomes*. Jan 2011;4(1):122-8. doi:10.1161/CIRCOUTCOMES.110.958397
16. Strategy and Planning Division of Office of the Permanent Secretary Ministry of Public Health. *Public Health Statistics* Ministry of Public Health; 2019.
17. Drug and Medical Supply Information Center MoPH. Reference price. Ministry of Public Health. Accessed 15 May 2022. http://dmsic.moph.go.th/index/drugsearch/1

18. Kotirum S, Chongmelaxme B, N C. A cost-utility analysis of dabigatran, enoxaparin, and usual care for venous thromboprophylaxis after hip or knee replacement surgery in Thailand. *J Thromb Thrombolysis*. Feb 2017;43(2):252-262. doi:10.1007/s11239-016-1433-5
19. Rattanachotphanit T, Limwattananon C, Waleekhachonloet O, Limwattananon P, K S. Cost-Effectiveness Analysis of Direct-Acting Oral Anticoagulants for Stroke Prevention in Thai Patients with Non-Valvular Atrial Fibrillation and a High Risk of Bleeding. *Pharmacoeconomics*. Feb 2019;37(2):279-289. doi:10.1007/s40273-018-0741-3
20. Ministry of Public Heath Thailand. *Thai Standard Costs of Public Health Services*. Ministry of Public Heath 2019.
21. Health Intervention and Technology Assessment: HITAP Ministry of Public Health. Standard Cost List for Heath Technology Assessment. Updated 2010. Accessed 15 May, 2022. https://costingmenu.hitap.net/
22. Pattanaphesaj J, Thavorncharoensap M, Ramos-Goñi JM, Tongsiri S, Ingsrisawang L, Y T. The EQ-5D-5L Valuation study in Thailand. *Expert Rev Pharmacoecon Outcomes Res*. Oct 2018;18(5):551-558. doi:10.1080/14737167.2018.1494574
23. Locadia M, Bossuyt PM, Stalmeier PF, et al. Treatment of venous thromboembolism with vitamin K antagonists: patients' health state valuations and treatment preferences. *Thromb Haemost*. Dec 2004;92(6):1336-41. doi:10.1160/TH04-02-0075
24. Christensen MC, Mayer S, JM F. Quality of life after intracerebral hemorrhage: results of the Factor Seven for Acute Hemorrhagic Stroke (FAST) trial. *Stroke*. May 2009;40(5):1677-82. doi:10.1161/STROKEAHA.108.538967
25. Ghofrani HA, D'Armini AM, Grimminger F, et al. Riociguat for the treatment of chronic thromboembolic pulmonary hypertension. *N Engl J Med*. 2013;369(4):319-29. doi:10.1056/NEJMoa1209657
26. Gage BF, Cardinalli AB, DK O. The effect of stroke and stroke prophylaxis with aspirin or warfarin on quality of life. *Arch Intern Med*. 1996;156(16):1829-36.
27. Law Y, Chan YC, Cheng SWK. Epidemiological updates of venous thromboembolism in a Chinese population. *Asian J Surg*. Mar 2018;41(2):176-182. doi:10.1016/j.asjsur.2016.11.005
28. Jun M, Lix LM, Durand M, et al. Comparative safety of direct oral anticoagulants and warfarin in venous thromboembolism: multicentre, population based, observational study. *BMJ*. Oct 17 2017;359:j4323. doi:10.1136/bmj.j4323
